# Liraglutide improved the reproductive function of obese mice by upregulating the testicular AC3/cAMP/PKA pathway

**DOI:** 10.1186/s12958-024-01202-0

**Published:** 2024-03-20

**Authors:** Ruibing Qi, Yuzhen Liang, Jinming Yu, Bing Chen, Jiaqin Jiang, Xingye Wu, Wensheng Lu, Zhengming Li

**Affiliations:** 1 Department of Endocrinology and Metabolism, Guangxi Academy of Medical Sciences and the People’s Hospital of Guangxi Zhuang Autonomous Region, Nanning, 530021 China; 2 Department of Endocrinology and Metabolism, Wuming Hospital of Guangxi Medical University, Nanning, 530199 China; 3 Department of Endocrinology, Second Affiliated Hospital of Guangxi Medical University, Nanning, 530007 China

**Keywords:** Glucagon-like peptide-1, Liraglutide, Obesity, Adenylyl cyclase 3, Sex hormone

## Abstract

**Background:**

The incidence of male reproductive dysfunction is increasing annually, and many studies have shown that obesity can cause severe harm to male reproductive function. The mechanism of male reproductive dysfunction caused by obesity is unclear, and there is no ideal treatment. Identification of effective therapeutic drugs and elucidation of the molecular mechanism involved in male reproductive health are meaningful. In this study, we investigated the effects of the GLP-1 receptor agonist liraglutide on sex hormones, semen quality, and testicular AC3/cAMP/PKA levels in high-fat-diet-induced obese mice.

**Methods:**

Obese mice and their lean littermates were treated with liraglutide or saline for 12 weeks. Body weight was measured weekly. Fasting blood glucose (FBG) was measured using a blood glucose test strip. The serum levels of insulin (INS), luteinizing hormone (LH), follicle stimulating hormone (FSH), testosterone (T), free testosterone (F-TESTO), estradiol (E2), and sex hormone binding globulin (SHBG) were detected using ELISA. The sperm morphology and sperm count were observed after Pap staining. The mRNA and protein expression levels of testicular GLP-1R and AC3 were measured by RT-qPCR and Western blot, respectively. Testicular cAMP levels and PKA activity were detected using ELISA.

**Results:**

Liraglutide treatment can decrease body weight, FBG, INS, HOMA-IR, E2 and SHBG levels; increase LH, FSH, T, and F-TESTO levels; increase sperm count; decrease the sperm abnormality rate; and increase GLP-1R and AC3 expression levels and cAMP levels and PKA activity in testicular tissue.

**Conclusions:**

Liraglutide can improve the sex hormone levels and semen quality of obese male mice. In addition to its weight loss effect, liraglutide can improve the reproductive function of obese male mice, which may also be related to the upregulation of AC3/cAMP/PKA pathway in the testis. This work lays the groundwork for future clinical studies.

## Introduction

With the rapid development of the global economy and improvements in living standards, obesity has become a significant health problem in the field of global public health. In 2020, the adult obesity rate in China was 50%, and the child obesity rate was 29% [1]. In recent years, the incidence of obesity in our country has been increasing, and obesity has attracted the attention of people from all walks of life. Notably, obesity has become the focus of attention not because of obesity itself but because it increases the risk of hypertension, diabetes, cardiovascular disease, sleep apnea syndrome, respiratory diseases, osteoarthritis, cancer, and other diseases [2]. The Infertility Prevalence Estimate (1990–2021) published by the World Health Organization (WHO) reports that approximately 17.5% of adults (approximately 1/6 of the population) worldwide are affected by infertility, and according to incomplete statistics, male factors contribute to 50% of infertility cases. In recent years, domestic and foreign studies have shown that obesity is an important risk factor leading to male infertility [2, 3]. Increasing evidence shows that obesity affects semen concentration and sperm motility and reduces semen quality [4]. However, the mechanisms of male reproductive disorders caused by obesity have not been fully elucidated, and there is no ideal medicine available for this disease; thus, it is urgently needed. Recent studies have shown that the AC3 gene is closely related to obesity. One obesity locus identified by genome-wide association studies is located within or near the AC3 gene [5]. The AC3 gene encodes the membrane-associated enzyme AC3, which catalyzes cAMP synthesis via adenosine triphosphate (ATP) [6]. Numerous studies have shown that cAMP and PKA play essential roles in sperm formation, motility, and capacitation [7, 8].

Liraglutide is a glucagon-like peptide-1 receptor agonist (GLP-1Ra) that shares 97% sequence homology with human GLP-1. Unlike natural GLP-1, liraglutide has greater enzymatic stability against DPP-IV, and its hypoglycemic and weight reduction effects have been recognized. Our previous studies have shown that liraglutide treatment can improve nonalcoholic fatty liver in obese mice and increase hepatic AC3/cAMP/PKA levels in obese mice [9–11]. There are very few studies on the relationship between GLP-1Ra and sex hormone levels and sperm morphology in obese male mice, and there are no reports on the effects of GLP-1Ra on testicular AC3/cAMP/PKA. Therefore, in this study, we established a mouse model of obesity to investigate the effects of the GLP-1Ra agonist liraglutide on sex hormone levels, sperm quality, and testicular AC3/cAMP/PKA in obese male mice to explore whether GLP-1Ra can improve the reproductive function of obese male mice and to provide new ideas for the clinical relief of reproductive dysfunction in obese individuals.

## Materials and methods

### Laboratory animals

Twenty-four SPF-grade 6-week-old male C57BL/6 J mice were purchased from Gempharmatech Co., Ltd. (animal use license number: SCXK(Su) 2018–0008).

### Principal reagents

High-fat feed (79% ordinary feed, 10% lard, 10% egg yolk powder, and 1% cholesterol) was purchased from Xietong Organism. Liraglutide was kindly provided by Novo Nordisk (Bagsvaerd, Denmark). Mouse insulin kits, mouse luteinizing hormone kits, mouse follicle-stimulating hormone kits, mouse testosterone kits, free testosterone kits, mouse estradiol kits, BCA protein quantification kits, and cAMP (cyclic adenosine monophosphate) ELISA Kits were purchased from Elabscience. A mouse sex hormone binding globulin kit and mouse protein kinase A kit were purchased from Jiangsu Enzyme Free Industrial Co., Ltd. TRIzol Reagent and an ultrapure RNA extraction kit were purchased from CWBIO. HiScript II Q RT SuperMix for qPCR and ChamQ Universal SYBR qPCR Master Mix were purchased from Vazyme. The 50 × TAE buffer, 30% acrylamide (PAGE Pre-Solution), 1 M Tris–HCl buffer (pH = 6.8), 1.5 M Tris–HCl buffer (pH = 8.8) and bovine serum albumin (BSA) were purchased from Solarbio. The 6 × DNA loading buffer was purchased from TRANS. A 50-bp DNA ladder was purchased from Tiangen. GSafe Red plus nucleic acid dye was purchased from GLPBIO. Agarose powder was purchased from Invitrogen. RIPA cell lysis buffer and sealed skim milk powder were purchased from Applygen. PVDF membranes were purchased from Millipore. The luminescent solution was purchased from Thermo Fisher Scientific. Mouse anti-beta-actin was purchased from TransGen Biotech. Horseradish peroxidase-conjugated goat anti-mouse IgG and horseradish peroxidase-conjugated goat anti-rabbit IgG were purchased from Servicebio. Rabbit anti-AC3 was purchased from Proteintech. Rabbit anti-GLP-1R was purchased from Bioss.

### Main methods

#### Experimental grouping and treatment

The animal experiment protocol of this study was approved by the ethical review of the Medical Ethics Committee of the Second Affiliated Hospital of Guangxi Medical University (Approval No. KY-0150 in 2020) (Approval No. KY-0894 in 2023).

Six-week-old mice (male) were adaptively fed for 1 week. The mice were then randomly divided into routine and high-fat diet groups, with 12 mice in each group. The mice in each group drank and ate freely and were kept in cages in standard animal rooms with light and darkness for 12 h, a temperature of ~ 20–26 °C, and a humidity of 40% ~ 70%. Body weights were measured every week. The average body weight of the obese mice was 20% greater than that of the normal mice after 10 weeks, indicating that the obesity model was successfully established. Subsequently, each group was randomly divided into 2 subgroups according to treatment drugs: the normal + saline group, normal + liraglutide group, obesity + saline group, and obesity + liraglutide group. The liraglutide groups were given a subcutaneous injection of liraglutide at 100 µg/(kg·d), and the saline groups were given an injection of an equal volume of 0.9% sodium chloride. The body weights of the mice were recorded weekly. Fasting blood glucose (FBG) was measured at week 12 of drug treatment, and mouse tail vein blood was collected for follow-up detection of sex hormones. The mice were killed by cervical dislocation, and the epididymides and testes were dissected quickly.

#### Blood glucose

FBG was measured in the 12th week after drug treatment. All the mice were fasted for 8 h. Blood was taken from the tail vein of the mice, and FBG levels were measured via glucose test paper.

#### Enzyme-linked immunosorbent assay (ELISA)

INS, LH, FSH, T, F-TESTO, E2, and SHBG levels in mouse serum, as well as testicular tissue cAMP levels and protein kinase A (PKA) activity were measured according to the kits’ instructions.

#### Determination of sperm count and sperm morphology

After the mice were killed, the epididymides were immediately dissected and placed in 1 mL of 0.9% NaCl (preheated at 37 °C). The mixture was incubated for approximately 30 min, after which the semen was routinely coated. The morphology of the spermatozoa was observed under a light microscope (CX43, Olympus), and the spermatozoa were counted in terms of density and viability.

Fresh semen was washed, prepared, and then treated with a sperm morphology staining kit (Pap method). After fixation in a 90% ethanol solution, the samples were rehydrated using a series of graded ethanol solutions by passing from high to low (80%, 70%, and 50%) for 1 min per gradient. Then, the sections were washed with distilled water for 1 min, stained with hematoxylin solution for 3–5 min, differentiated with acid ethanol differentiation solution for approximately 4–5 s after rinsing, observed under a microscope to the appropriate degree, and stained for 4 min in bluing solution. After rinsing, the samples were dehydrated in 50%, 70%, 80%, and 95% ethanol series for 1 min for each gradient, after which the samples were incubated with orange G6 staining solution for 2 min. After rinsing and dewatering with ethanol, the sperm morphology was observed. The types of sperm abnormalities were observed, and the percentage of sperm abnormalities was determined.

#### Quantitative reverse transcription PCR (RT‒qPCR)

The primers for the target genes were synthesized according to the mouse gene sequences in GenBank (Table 1). Total RNA was extracted from testicular tissue homogenates using TRIzol reagent, and mRNA was extracted using an ultrapure RNA extraction kit. The mRNA concentration and purity (OD260/OD280) were determined via ultraviolet spectrophotometry, and cDNA was synthesized via reverse transcription according to the RNA reverse transcription kit instructions. qPCR was performed by the SYBR Green fluorescence technique in a fluorescent PCR apparatus (CFX Connect™ Real-Time, Bio-Rad Laboratories) under the following conditions: 95 °C for 10 min; 40 cycles of 95 °C for 10 s, 58 °C for 30 s, and 72 °C for 30 s. β-Actin was used as the internal reference control. The Ct value (the number of cycles at which the fluorescence intensity reached the threshold) was used as the statistical parameter, and the relative gene expression was calculated according to the 2^^−△△Ct^ method.

**Table 1:** Primer sequences

Primer name	Forward primer (5’-3’)	Reverse primer (5’-3’)
β-actin	AGGGAAATCGTGCGTGAC	CATACCCAAGAAGGAAGGCT
AC3	TTTACAAGCTCCAGCAAGGAGG	ACATTGACCGTATTGCCCCA
GLR-1R	TCTTTGCTATCGGCGTCAACT	GCAGTACAGGATAGCCACCA

#### Western blot

Approximately 50 mg of testicular tissue was added to 1 ml of RIPA cell lysis buffer and tiny steel beads for grinding, after which the mixture was fully ground using a grinder (Tiss-12, Shanghai Jingxin Industrial Development Co., Ltd.). The mixture was then centrifuged at 12,000 r/min for 10 min at 4 °C, after which the supernatant was collected. The protein concentration was measured using the BCA method. Ten micrograms of protein was subjected to SDS‒PAGE, and the protein bands were transferred to a PVDF membrane using the wet transfer method. The blot was blocked with 3% skim milk powder for 1.5 h. Then, a polyclonal GLP-1R antibody, a polyclonal AC3 antibody, and a polyclonal cAMP antibody were added overnight at 4 °C on a shaking platform. After the membrane was washed with TBST three times, it was incubated with horseradish peroxidase-conjugated goat anti-rabbit IgG for 2 h at room temperature on a shaking platform. Finally, the membrane was developed using the ECL method. The images were then captured using the ChemiDoxTM XRS + Gel Imaging System and analyzed using ImageJ software in grayscale. Finally, the images were analyzed in grayscale using ImageJ software.

### Statistical analysis

All the statistical analyses were performed using SPSS 26.0 software, and all the data are expressed as the means ± standard deviations (mean ± SD). If the variance was homogeneous, one-way ANOVA was used to compare the data of multiple groups, and the LSD method was used to compare the data of two groups. If the variance was not homogeneous, the Welch test was used to compare the data of multiple groups. Tamhane’s T2 was used to compare the data from two-way comparisons between groups, and P < 0.05 was considered to indicate statistical significance.

## Results

### Liraglutide reduced body weight and insulin resistance in obese male mice

The body weight, FBG, INS, and HOMA-IR values of obese mice were significantly greater than those of normal mice, and liraglutide significantly reduced the weight, FBG, INS, and HOMA-IR values of obese mice (Table 2).

**Table 2:** Body weight and insulin resistance of the mice in each group

	**Normal + saline**	**Obesity + saline**	**Normal + liraglutide**	**Obesity + liraglutide**
Body weight(g)	28.10 ± 1.08	34.78 ± 1.88^###^	27.23 ± 0.99	29.69 ± 0.77***^###^
FBG (mmol/L)	6.07 ± 0.97	11.45 ± 1.04^##^	5.54 ± 0.70	9.28 ± 0.99***^###^
INS (mIU/L)	7.05 ± 0.55	8.78 ± 1.13^##^	6.18 ± 0.54	5.05 ± 0.67***^###^
HOMA-IR	1.91 ± 0.37	4.64 ± 0.55^###^	1.60 ± 0.16	2.13 ± 0.33***

Values are the mean ± SD (*n* = 6 per group). The means of multiple samples of mouse body weight and insulin (INS) data were compared using one-way ANOVA, and two-group comparisons were made using the LSD method. Mouse fasting blood glucose (FBG) and the homeostasis model assessment of insulin resistance (HOMA-IR) were compared using the Welch test for mean comparisons between samples of multiple groups and Tamhane’s T2 method for two-group comparisons. Between saline and liraglutide, ***p < 0.001; between normal and obese, ^##^*p* < 0.01, ^###^*p* < 0.001

### Liraglutide improves sex hormone levels in obese mice

**Fig. 1:**
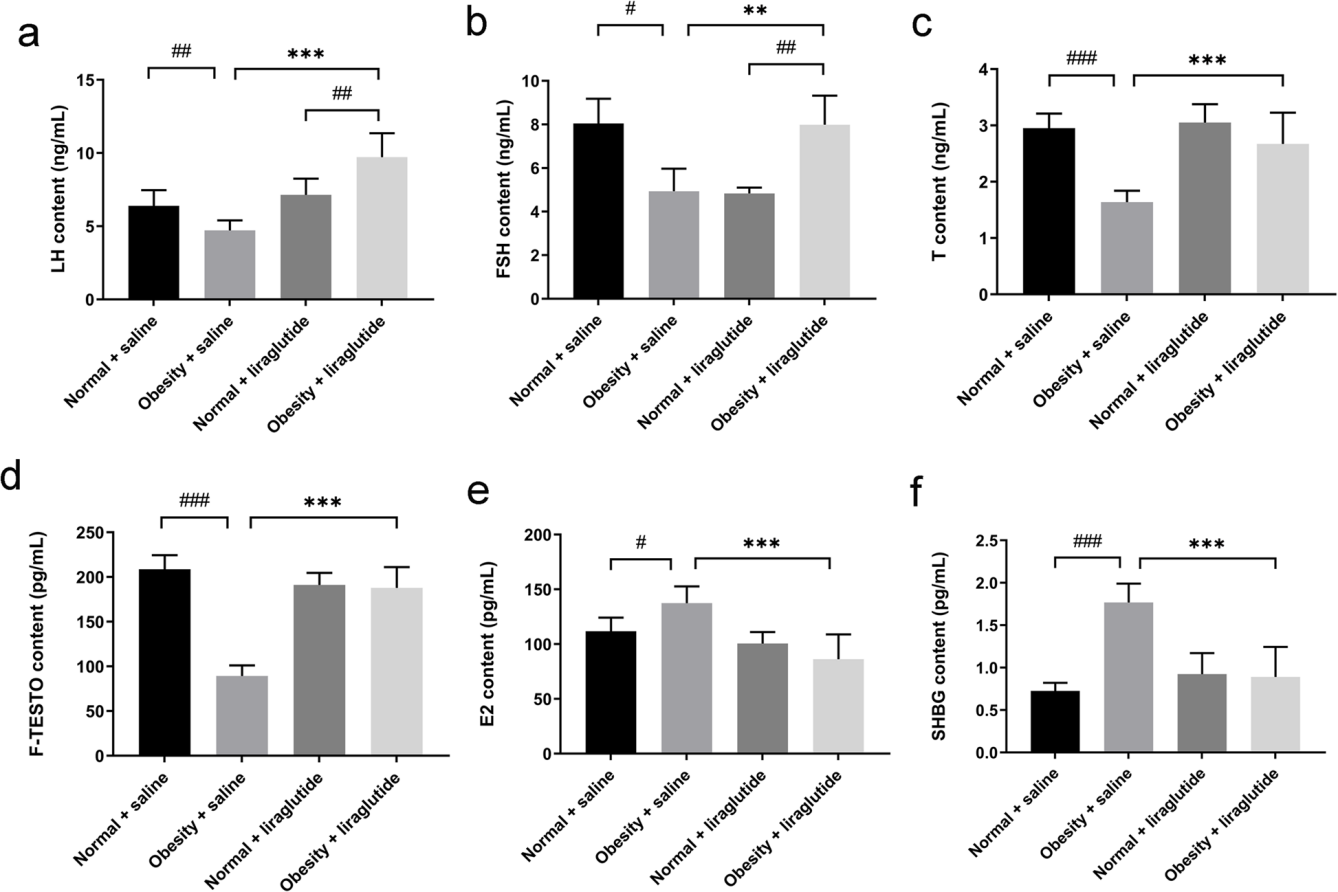
Sex hormone profiles of the mice in each group. Notes:Values are the mean ± SD (*n* = 3 per group, repeat 3 times). The means of mouse luteinizing hormone (LH), testosterone (T), free testosterone (F-TESTO), estradiol (E2), and sex hormone binding globulin (SHBG) levels were compared using one-way ANOVA, and two-group comparisons were made using the LSD method. Mouse follicle-stimulating hormone (FSH) data were compared using the Welch test for mean comparisons between samples of multiple groups and Tamhane’s T2 method for two-group comparisons. Between saline and liraglutide, **p < 0.01, ***p < 0.001; between normal and obese, ^#^*p* < 0.05, ^##^*p* < 0.01, ^###^*p* < 0.001

The serum levels of LH, FSH, T, and F-TESTO were significantly lower in the obese group than in the normal group, and the E2 and SHBG levels were significantly greater than those in the normal group. Liraglutide significantly increased the serum levels of LH, FSH, T, and F-TESTO and significantly decreased the levels of E2 and SHBG (Fig. 1).

### Effects of liraglutide on the sperm of obese male mice

**Fig. 2:**
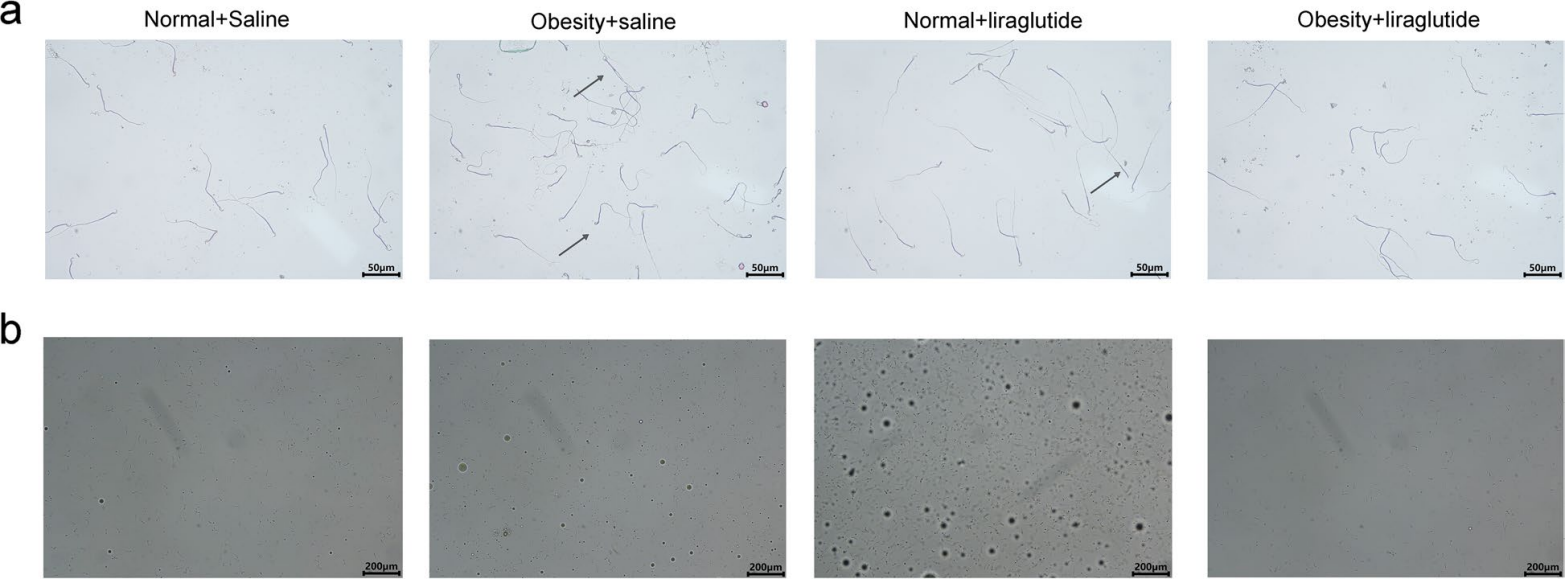
Sperm of mice in each group and plot of Pap staining of mouse sperm in each group (arrowheads indicate malformed sperm)

The sperm count of mice in the obese groups was significantly lower than that of mice in the normal groups, and the sperm abnormality species and sperm abnormality rates were significantly greater than those of normal mice. Liraglutide significantly increased the sperm count and decreased the number of abnormal sperm species and the percentage of abnormal sperm in obese mice (Table 3, Fig. 2).

**Table 3:** Number of sperm in each group of mice

	**Normal + saline**	**Obesity + saline**	**Normal + liraglutide**	**Obesity + liraglutide**
Sperm count (piece)	92 × 10^4 ± 5.9 × 10^4	30 × 10^4 ± 6.8 × 10^4^###^	150 × 10^4 ± 15 × 10^4**	66 × 10^4 ± 9.2 × 10^4**^###^
Sperm abnormal rate (%)	25 ± 1.4	52 ± 3.5^###^	18 ± 1.4*	31 ± 2.8***^##^

Values are the mean ± SD (*n* = 3 per group). One-way ANOVA was used to compare means between multiple samples, and the LSD method was used for two-group comparisons. Between saline and liraglutide, *p < 0.05, **p < 0.01, ***p < 0.001; between normal and obese, ^##^*p* < 0.01, ^###^*p* < 0.001

### Effects of liraglutide on GLP-1R, AC3, cAMP, and PKA activity in the testes of obese mice

**Fig. 3:**
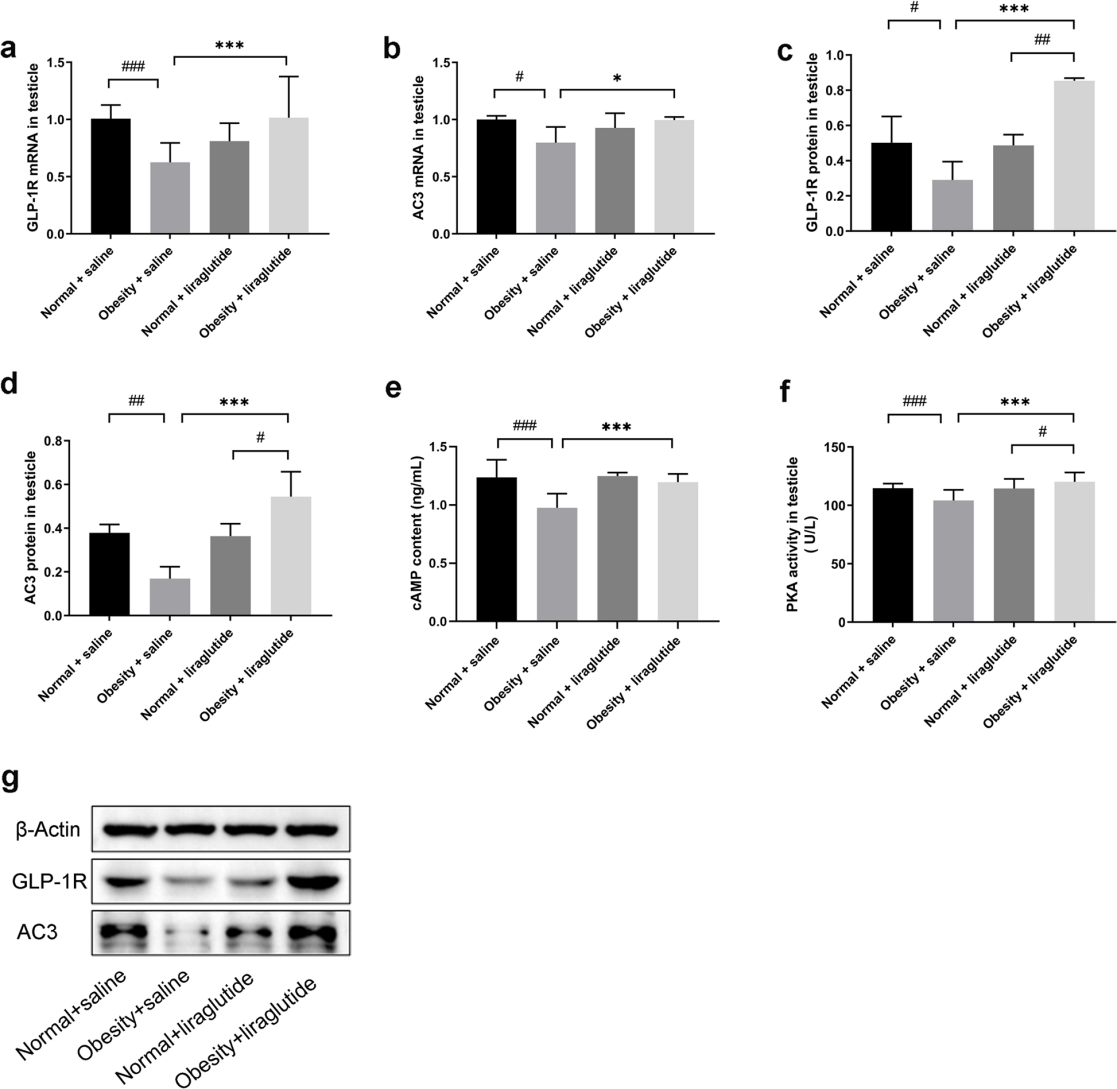
Glucagon-like peptide-1 receptor (GLP-1R) and adenylate cyclase-3 (AC3) expression at the mRNA levels and protein levels, and cyclic adenosine monophosphate (cAMP) levels and Protein kinase A (PKA) activity in testicular tissues of mice in each group. Notes: Values are the mean ± SD (*n* = 3 per group, repeat 3 times). The means of multiple samples of mouse testis tissue GLP-1R mRNA expression data and GLP-1R, AC3 protein expression data, as well as cAMP levels and PKA activity were compared using one-way ANOVA, and two-group comparisons were made using the LSD method. Mouse testis tissue AC3 mRNA data were compared using the Welch test for mean comparisons between samples of multiple groups and Tamhane’s T2 method for two-group comparisons. Between saline and liraglutide, *p < 0.05, ***p < 0.001; between normal and obese, #*p* < 0.05, ##*p* < 0.01, ###*p* < 0.001

Testicular tissue from obese mice had significantly lower GLP-1R and AC3 mRNA levels than testicular tissue from normal mice. Liraglutide significantly upregulated the mRNA expression of GLP-1R and AC3 in the testes of obese mice. (Fig. 3a-b). Testicular tissue from obese mice had significantly lower GLP-1R and AC3 protein levels than testicular tissue from normal mice. Liraglutide significantly upregulated the protein expression of GLP-1R and AC3 in the testes of obese mice (Fig. 3c-d). The results of testicular cAMP levels and PKA activity analysis in mice by ELISA showed that cAMP levels and PKA activity in obese mice were significantly lower than that in normal mice, and liraglutide significantly increased cAMP levels and PKA activity (Fig. 3e–f).

## Discussion

The number of cases of male reproductive dysfunction is increasing each year. In recent years, numerous studies have shown that overweight and obesity are also important causes of male infertility and that obesity can cause serious harm to male reproductive function [12, 13]. Some studies have shown that obesity affects semen quality and leads to male reproductive dysfunction [14]. The mechanism of male reproductive dysfunction caused by obesity is unknown, and no ideal therapeutic drugs exist. It is essential to search for effective therapeutic drugs and to study the molecular mechanisms involved in male reproductive health.

Liraglutide is a GLP-1 receptor agonist, and its glucose-lowering and weight-loss effects have been well recognized. In this study, obese mice were fed with high-fat diet and treated with liraglutide after successful modeling. The results showed that liraglutide could reduce body weight, FBG, fasting insulin and HOMA-IR in obese mice. There were no significant differences in body weight, FBG, fasting insulin or HOMA-IR between the two groups of normal mice. Our results confirmed the glucose concentration-dependent effect of liraglutide on insulin secretion and hypoglycemia [15]. At present, HOMA-IR is widely used to evaluate the degree of insulin resistance, and the improvement of insulin resistance is largely related to weight loss. It is generally believed that the effect of liraglutide on weight loss is related to the baseline body weight. Many studies have shown that liraglutide has no significant effect on normal weight [16, 17]. The glucose concentration-dependent effect of liraglutide on postprandial insulin secretion has been recognized, but there are few studies on its effect on fasting insulin levels. Our results suggest that liraglutide can reduce fasting insulin levels in obese mice. We believe that this effect is related to its improvement of insulin resistance, which is also consistent with the findings of previous studies [18].

Obesity can affect male reproductive health in many ways, including through endocrine disorders, chronic low-grade inflammation, oxidative stress, and genetic factors. It is generally believed that obesity may disrupt the physiological balance of the hypothalamic-pituitary–gonadal (HPG) axis by affecting central and peripheral mechanisms, resulting in changes in the serum levels of FSH, LH, T, F-TESTO, and E2. Testosterone can act directly on target cells via androgen receptors or can be converted to dihydrotestosterone via 5α-reductase or to 17β-estradiol by the cytochrome P450 aromatase [19]. White adipose tissue expresses high levels of the cytochrome P450 aromatase [20]. The aromatization of testosterone in the peripheral adipose tissue of men with obesity leads to an increase in E2 levels, inhibits the HPG axis through negative feedback, and reduces the secretion of FSH and LH by the pituitary gland [21]. This study showed that FSH, LH, T, and F-TESTO levels in obese male mice were significantly lower than those in normal mice, and E2 levels were significantly greater than those in normal mice. Some scholars believe that the low T level in obese people may be caused by the decrease in SHBG binding activity caused by the increase in INS levels [21]. This leads to a relative increase in free testosterone and amplifies the effect of negative estradiol feedback through aromatization. Only free sex hormones are biologically active in the body. SHBG is synthesized in the liver and can specifically bind to circulating sex hormones (including testosterone and estrogen) to regulate serum sex hormone bioavailability [22], thus regulating hormone levels in the body. According to the study of sex hormones in women with obesity, obesity causes an increase in INS levels, which increases INS resistance, and INS resistance can inhibit the liver's production of SHBG, causing a decrease in SHBG, resulting in an increase in free testosterone levels and thus affecting fertility [23–26]. This study showed that the SHBG level in obese male mice was greater than that in normal mice. Our results are inconsistent with previously observed declines in SHBG in women with obesity, but the relationship between SHBG and testosterone has not been reported in current studies of sex hormones in men with obesity. More research is needed to determine whether the relationship between SHBG and testosterone differs between the sexes. We speculate that the increase in the SHBG content in obese male mice was one of the reasons for the decrease in free testosterone levels and that liraglutide intervention can reduce SHBG and increase free testosterone. In this study, liraglutide could significantly increase the levels of LH, FSH, T and F-TESTO in obese mice and significantly decreased the levels of E2 and SHBG in obese mice, while liraglutide had no significant effect on the levels of LH, FSH, T, F-TESTO, E2 or SHBG in normal mice. Studies have shown that continuous intravenous infusion of GLP-1 for 500 min does not affect the levels of LH, FSH or T in healthy young men with normal weights [27]. Although the effects of liraglutide on the LH and FSH levels in normal mice were not statistically significant, we observed that liraglutide had different effects on the LH and FSH levels in normal mice. After treatment, the LH level of normal mice tended to increase, while the FSH level tended to decrease. Kisspeptin regulates the function of the HPG axis, which is upstream of GnRH, and GnRH regulates the secretion of LH and FSH. Although LH and FSH are regulated by GnRH, they play different roles. LH can stimulate Leydig cells to produce testosterone, and FSH activates Sertoli cells, which nourish developing sperm cells at different stages of spermatogenesis. In healthy men, injection of kisspeptin-10 or GnRH significantly increased the blood LH levels, but injection of kisspeptin-10 had no effect on the blood FSH levels [28]. The effect of GnRH injection on FSH was not mentioned in this paper [28]. In animal experiments, intracerebroventricular injection of GLP-1 rapidly increased the concentration of plasma LH in rats but had no effect on FSH [29]. We consider that the ability of liraglutide to improve sex hormone levels can be achieved through its weight loss effect, but weight loss may not be the only factor involved. However, additional studies are needed to determine the effects of liraglutide on LH and FSH.

Some evidence suggests that human Leydig cells, Sertoli cells, and germ cells all express GLP-1 receptors, suggesting that GLP-1RA may have a direct effect on testicular tissue [30, 31]. GLP-1 plays different roles in different tissues by acting on the GLP-1 receptor on the cell membrane, and approximately 70% of the biological functions of GLP-1 involve the cAMP signaling pathway [32]. After GLP-1 binds to G protein-coupled receptors, adenylate cyclase is activated to convert ATP into cAMP, resulting in cAMP-dependent activation of secondary messenger pathways such as the PKA and Epac pathways [33]. The ACs family consists of 10 members: AC1-9 and soluble adenylate cyclase (commonly known as sAC or AC10). AC1-9 are the main effector enzymes of the G protein-coupled receptors (GPCRs), and sAC (or AC10) is regulated by bicarbonate and calcium rather than by G protein [34]. AC3 was initially found in the cilia of olfactory cells and plays a vital role in olfactory conduction [35]. The Chinese scholar Zhou Yanfen has shown that AC3 deletion can downregulate the expression of olfactory receptor genes [36]. However, olfaction plays a critical role in mating behavior, and Boehm et al. reported that AC3-/- mice were incapable of reproduction [37]. Both Defer and Gautier-Courteille reported AC3 expression in semen [38, 39]. Gautier-Courteille identified immunoreactive peptides with AC3 characteristics in testicular and germ cell extracts. Immunofluorescence localization in the curved tubule indicated that AC3 in sperm cells, including round sperm cells in the cap stage and mature elongated sperm cells, exhibited strong signals at different stages of differentiation. Furthermore, sperm from AC3-/- mice exhibit reduced motility and impaired fertilization in vitro [40]. This study also confirmed that the GLP-1 receptor and AC3 are expressed in the mouse testis. In this study, we detected the expression of GLP-1R and AC3 mRNA and protein in mouse testes, but we did not distinguish the specific germ cells.

One of the leading causes of male infertility is defective sperm motility and capacitation. To achieve fertilization, sperm need to undergo a complex series of activation processes. Haploid sperm lack functional ribosomes, the endoplasmic reticulum, the Golgi apparatus, etc. Therefore, sperm are highly dependent on the secondary messenger system. cAMP formation catalyzed by AC3 is an essential regulatory factor. As an important secondary messenger, it regulates various signaling pathways and is crucial for regulating sperm motility, maturation, and capacitation. Both cAMP and its targeted receptors regulate various signaling pathways, including intracellular pH increase and protein tyrosine phosphorylation, which in turn regulate capacitation processes and acrosome reactions [41]. The pH within male germ cells plays a vital role in sperm motility, and the sodium-hydrogen exchanger NHE primarily maintains the intracellular pH balance. Studies have shown that the motility of NHE-/- sperm is strongly decreased, possibly leading to complete male infertility. Moreover, the addition of cAMP analogs can ameliorate sperm motility and fertility defects [42]. cAMP and PKA are closely related to spermatogenesis and sperm function. cAMP-dependent PKA is the main downstream effector of cAMP signaling in sperm. During capacitation, the increase in cAMP activates cAMP-dependent PKA, resulting in the phosphorylation of a large amount of sperm protein, especially the axoneme protein, by PKA, leading to the induction of flagellar motility. Modifying protein structure and function promotes the completion of a large amount of sperm maturation [41, 43]. Recent studies have confirmed the central role of cAMP in regulating sperm movement through flagellar movement and velocity analysis. cAMP is a prerequisite for inducing lateral head displacement and flagellar movement in the XY and Z planes, resulting in an increase in swimming speed [41]. In vitro studies have shown that when PKA-Cα is defective, in addition to motor defects, it also exacerbates sperm head distortion [43]. This study showed that the sperm count of obese mice was lower than that of normal mice, and the types and rates of sperm abnormalities were greater than those of normal mice. Liraglutide treatment significantly increased the sperm count of obese mice and reduced the types and percentages of sperm abnormalities. In the present study, the mRNA and protein expression levels of GLP-1R and AC3, as well as cAMP levels and the activity of PKA in the testes of normal and obese mice were compared, and the effects of liraglutide treatment on the above indices were evaluated. Our data showed that the mRNA and protein expression levels of GLP-1R and AC3 as well as cAMP levels and the activity of PKA in the testes of obese mice were significantly lower than those in the testes of normal mice. Liraglutide intervention upregulated the mRNA and protein expression of GLP-1R and AC3, and increased the cAMP levels and activity of PKA in the testes of obese mice. Therefore, we speculate that liraglutide may play a positive role in the reproductive function of obese mice through regulating the testis AC3/cAMP/PKA pathway, but further studies in knockout mice and at the cellular or molecular level are needed to reveal the underlying mechanisms involved.

## Conclusions

This study showed that liraglutide can improve the sex hormone levels and semen quality of obese male mice. In addition to its weight loss effect, liraglutide can improve the reproductive function of obese male mice, which may also be related to the upregulation of the AC3/cAMP/PKA pathway in the testis; further research is needed to determine the underlying mechanism involved.

## Data Availability

No datasets were generated or analysed during the current study.
